# Commentary: Uterine Microbiota: Residents, Tourists, or Invaders?

**DOI:** 10.3389/fimmu.2018.01874

**Published:** 2018-08-24

**Authors:** Signe Altmäe

**Affiliations:** ^1^Department of Biochemistry and Molecular Biology, Faculty of Sciences, University of Granada, Granada, Spain; ^2^Competence Centre on Health Technologies, Tartu, Estonia

**Keywords:** endometrium, menstrual cycle, microbiome, microbiota, seminovaginal microbiota, uterus

The recently published review by Baker et al. summarizes the current status of uterine microbiota with the aim to promote research priorities and discussion on this novel research field (1). The authors are to be congratulated on this much anticipated review as microbiota in the uterus is one increasing research area, though poorly investigated microbial niche relative to other organs. However, emerging evidence is beginning to indicate that the uterine microbiota has important implications for female (reproductive) health and disease, and it is becoming evident that the concept of sterile uterus is outworn, although the true core uterine microbiota still needs to be assessed.

In their comprehensive review, Baker et al. present established and putative bacterial transmission routes between uterine microbiota and distal sites, where they highlight (a) hematogenous spread of bacteria through either oral or gut route, (b) ascension of bacteria through the cervix, and (c) other routes such as retrograde spread through fallopian tubes, assisted reproductive technology-related procedures or insertion/removal of intrauterine devices together with its potential aid in ascension through the “tails” of the device (1).

There is, however, another important bacterial transmission route that has high potential to influence uterine microbiota that the authors have missed to present—the seminal microbiota. Even before the era of 16S RNA analysis, it was postulated that “it is difficult to envision that a mucosa continuously exposed to microorganisms present in the lower genital tract and that is regularly invaded by sperm that can carry microorganisms into the endometrial cavity may be free of bacteria” (2). Indeed, a term “complementary seminovaginal microbiota” has been recently proposed (3). Recent studies are demonstrating that bacteria are shared among partners and that partners influence the species composition of each other’s reproductive tract microbiota (3–6), with sexual debut and activity having significant impact (4, 5). In line with sexual activities and hematogenous spread of bacteria emanating from the gut and oral microbiota, oral and anal sex can influence the microorganismal continuum, as is known with different diseases caused by sexually transmissible pathogens (e.g., oral lesions, proctitis, proctocolitis, and enteritis) (7, 8). Interestingly, the placental microbiome resembles that of the oral cavity more than that of the gut or even vagina (9). In short, semen serves as a perfect medium for the transmission of microorganisms (being slightly basic and enriched with carbohydrates it creates an ideal habitat for microorganisms), which should be considered as one important route of microorganismal tourism or invasion, with potential to become residents in the uterus.

Furthermore, Baker et al. mention briefly in their review that bacterial seeding of the uterus has important ramifications on maternal–fetal transfer of microbiota and postnatal health (1). Also here the paternal contribution should be highlighted, as it is clear that male contribution to offspring is more than just the haploid genome complement in sperm. It has been recently proposed that fathers may transmit information *via* microbiota to their partners and progeny (10). Novel studies are providing knowledge of possible mechanisms of microbiota’s role on offspring, where the influence on methylome and transcriptome changes, and on microglia has been shown (11, 12).

There is, however, one aspect that needs to be clarified, as Baker et al. conclude in their review that it is not clear if the uterine microbiota changes during the menstrual cycle (1). The authors mention that the only study assessing uterine microbiota across two different time points of the menstrual cycle has been Moreno et al. (13). In that study, the uterine microbiome was similar at the two hormonal stages, but as the authors adequately conclude that these results should be viewed with some caution (13). Given the fact that hormonal changes influence vaginal microbiota (14, 15), that microbiota is influenced by hormones (16), and that the use of gonadotrophin-releasing hormone agonist resulted in a shift of uterine microbiome composition (17), one would expect that also uterine microbiota is influenced by sex hormones during natural menstrual cycle. Indeed, what Baker et al. have missed to present in their review, is the study results by Chen et al. (18), where microbiota continuum along the female reproductive tract on 95 women in the proliferative and secretory phases were studied. Operational taxonomy units that led to optimal classification between the two phases in the uterus included *Sphingobium* sp., *Propionibacterium acnes*, and *Carnobacterium* sp. Interestingly, *P. acnes*, that has previously been identified in the placenta and follicular fluid, was more abundant in the secretory phase uterus (18). Enrichment analysis identified different pathways associated with increased bacterial proliferation (pyrimidine and purine metabolism, and aminoacyl-tRNA biosynthesis) in the proliferative phase compared to the secretory phase (Table 1).

**Table 1:** Enrichment analysis of microbial KEGG pathways in the proliferative and the secretory phases in the endometrium from 80 reproductive-aged women (18) (adapted with permission from Nature Publishing Group).

Top microbial KEGG pathways enriched in the endometrium	

Proliferative phase	Secretory phase
Phosphotransferase system	ABC transporters
Pyrimidine metabolism	Porphyrin and chlorophyll metabolism
Purine metabolism	Glyoxylate and dicarboxylate metabolism
Aminoacyl-tRNA biosynthesis	Phenylalanine, tyrosine, and tryptophan biosynthesis
Galactose metabolism	Flagellar assembly
Homologous recombination	Lipopolysaccharide biosynthesis
Mismatch repair	Atrazine degradation
Base excision repair	Phenylalanine metabolism
Amino sugar and nucleotide sugar metabolism	Arginine and proline metabolism
Fructose and mannose metabolism	Benzoate degradation

*This work is licensed under the Creative Commons Attribution 4.0 International License. To view a copy of this license, visit http://creativecommons.org/licenses/by/4.0/or send a letter to Creative Commons, PO Box 1866, Mountain View, CA 94942, USA*.

In addition, there are two new studies published that support the concept of microbial changes throughout the menstrual cycle, where bacterial continuum between proliferative and secretory phases differed in (1) endometria from dysmenorrhea and menorrhagia patients (19) and (2) in the fallopian tubes (20). Clearly, more studies are required for identifying the “baseline” microbial continuum in the uterus, nevertheless the first studies are showing the uterine microbiota differences along the menstrual cycle. Furthermore, the importance of the microbiota in regulation of rhythmic biological changes has recently been proposed (21), and that fluctuating microbial community structures might direct hormonal changes (22), it is tempting to hypothesize that similar dynamics might be involved in the female menstrual cycle (23).

As it stands, the assessment of uterine microbiota suffers from many limitations, there is a need for functional studies as well as for well designed and larger sample cohorts to unravel the role of microorganisms (and not only bacteria but also viruses, fungi, microscopic eukaryotes, and archaea) in uterine health and pathologies. Nevertheless, the novel studies are indicating that microbiota is another piece in the complex mechanism contributing to the cogwheels of hormones and physiological adaptations that are required for successful embryo implantation and pregnancy.

## Author Contributions

SA conceived and wrote the manuscript.

## Conflict of Interest Statement

The author declares that the research was conducted in the absence of any commercial or financial relationships that could be constructed as a potential conflict of interest.
